# High-Density Lipoprotein and Heart Failure

**DOI:** 10.31083/j.rcm2411321

**Published:** 2023-11-21

**Authors:** Liyun Xing, Yixuan Liu, Jiayu Wang, Peiqing Tian, Ping Liu

**Affiliations:** ^1^Department of Cardiology, the Second Hospital of Shandong University, 250033 Jinan, Shandong, China; ^2^School of Clinical and Basic Medicine, Shandong First Medical University, 250117 Jinan, Shandong, China

**Keywords:** high-density lipoprotein, high-density lipoprotein cholesterol, heart failure, inflammation, apolipoprotein

## Abstract

The protective effect of high-density lipoprotein (HDL) on atherosclerosis is well known, and its mechanisms of action has been extensively studied. However, the impact of HDL on heart failure and its mechanisms are still controversial or unknown. The cardioprotective role of HDL may be reflected in its antioxidant, anti-inflammatory, anti-apoptotic, and endothelial function protection. In epidemiological studies, high-density lipoprotein cholesterol (HDL-C) levels have been negatively associated with heart failure (HF). The major protein component of HDL-C is apolipoprotein (Apo) A-I, while paraoxonase-1 (PON-1) is an essential mediator for many protective functions of HDL, and HDL may act through components like (Apo) A-I or PON-1 to delay heart failure progress. HDL can slow heart failure disease progression through parts like (Apo) A-I or PON-1. The potential causality between HDL and heart failure, the role of HDL in the pathogenesis of HF, and its interaction with C-reactive protein (CRP), triglycerides (TG), and monocytes in the process of heart failure have been briefly summarized and discussed in this article. HDL plays an important role in the pathogenesis, progression and treatment of HF.

## 1. Introduction

Heart failure (HF) is a complex clinical syndrome caused by a structural or functional disorder of the heart that results in impaired ventricular filling or blood expulsion, which occurs when the blood and oxygen delivered by the heart cannot support other body organs [1, 2]. HF is one of the leading causes of morbidity and mortality worldwide, making it more widely studied than other diseases. Epidemiological studies have shown that the prevalence of HF in the Western world is estimated at 2%, with an annual incidence of close to 5–10 per 1000 people. The majority of HF is 7% in the 75–84 age group and over 10% in the over-85 age group. The age-adjusted mortality rate five years after the onset of heart failure was 50% for men and 46% for women [3]. In terms of etiology, HF can be caused by various underlying heart diseases, and most cardiovascular diseases eventually develop into heart failure [4]. Most HF is caused by coronary heart disease (CHD). In China, more than 10 million people suffer from CHD [5]. More importantly, compared to other cardiovascular diseases, the signs and symptoms of HF are challenging to distinguish from a wide range of differential diagnoses, which makes its pathological mechanisms and clinical manifestations very interesting to study [6]. There are some differences in the prevalence of HF between males and females, as evidenced by the fact that HF is more frequent in males under 80 compared to females. Ischemic heart disease is more common in men suffering from HF, with reduced ejection fraction (HFrEF). However, hypertensive heart disease is a more common complication in older women with HF with preserved ejection fraction (HFpEF). In addition to hypertension, common complications in women with HF include diabetes, anemia, and thyroid disease. This suggests that heart failure in women is likely associated with obesity and a sedentary lifestyle [7]. Due to the extensive public health burden and complexity of HF, it is necessary to analyze its relevance from information obtained from clinical cases and tests.

In the general population, high-density lipoprotein cholesterol (HDL-C) has been found to substantially reduce both cardiovascular and non-cardiovascular mortality. Emerging researches suggest that the anti-inflammatory properties of high-density lipoprotein (HDL) may contribute to this beneficial effect. Conversely, there is evidence suggesting that inflammation can decrease both the levels and functionality of HDL-C [8]. Previous clinical trials have demonstrated that low-density lipoprotein cholesterol (LDL-C) and high levels of HDL-C are associated with a reduced incidence of CHD and improved prognosis in patients with HF [5] . A compelling body of evidence has revealed a significant negative correlation between HDL and HF. In light of this, we conducted a systematic review of the existing literature to comprehensively examine the mechanism and role of this biochemical marker in the pathogenesis, progression, and treatment of heart failure.

## 2. The Structure of High-Density Lipoprotein Cholesterol

HDLs exhibit a diverse composition consisting of multiple subgroups of lipid and lipoprotein particles characterized by varying sizes and surface charges in the bloodstream. This heterogeneity is a result of the remodeling process undergone by individual HDL particles, influenced by various plasma factors [9]. Despite this heterogeneity, all HDLs are composed of lipids and proteins, with phospholipids, unesterified cholesterol, and the primary protein (Apo) A-I [10] being particularly important in the mechanisms of action. The anti-inflammatory effects of HDL are manifested in increased prostacyclin release from smooth muscle cells via the cyclooxygenase 2 dependent pathway and decreased expression of endothelial cell adhesion molecules induced by cytokines [11]. HDL protects the survival and function of the organism through various overlapping mechanisms. For example, HDL related proteins and bioactive lipids directly activate some signal transduction pathways. HDL also acts indirectly by excreting cholesterol from cells, influencing cholesterol homeostasis, eliminate potential hazards through enzymes such as paraoxonase (PON), transporting cholesterol to the liver for biotransformation through pathways shared with reverse cholesterol transport, and excretion [9] . Over the past decades, many epidemiological studies have shown that plasma HDL-C is negatively associated with cardiovascular disease risk [12]. Sphingosine-1-phosphate (S1P) is a component of HDL, and an accumulating body of research has shown that S1P mediates many cardiovascular effects of HDL, including the ability to promote vasodilation, vasoconstriction, angiogenesis, prevent ischemia/reperfusion injury, and inhibit atherosclerosis [13]. In terms of molecular mechanisms, HDL exerts its effects by activating the G protein-coupled receptor and PI3 kinase (phosphoinositide 3-kinase signaling pathways) signaling pathways. A critical component involved in these processes is apolipoprotein M (Apo M), which acts as a chaperone for sphingosine-1-phosphate (S1P). Animal studies have demonstrated that Apo M plays a pivotal role in mediating S1P signaling, thereby promoting anti-inflammatory effects, enhancing cardiomyocyte survival, and improving endothelial function [14]. These findings highlight the significance of Apo M in the beneficial actions of HDL, emphasizing its potential as a therapeutic target for modulating inflammation and cardiovascular health.

## 3. The Role of HDL in Heart Failure

Over the past 30 years, lipid interventions have received much attention in preventing and treating CHD, a cause of heart failure. Despite this focus, limited data exists regarding the potential association between circulating levels of HDL and heart failure. Extensive epidemiological studies suggest low HDL and low (Apo) A-I levels may increase the risk of HF. In contrast, some controversy points to the possibility that coronary ischemia may be an important confounding factor in these observations [15]. Experimental studies indicate HDL exhibits cardioprotective effects through its antioxidant properties, as well as its ability to counter inflammation, apoptosis, and endothelial dysfunction [16]. Within the distribution of HDL subpopulations, small, dense, protein-rich HDL has shown antiatherosclerotic properties attributed to specific protein and lipid clusters. Several authors have demonstrated that high-density lipoprotein particles (HDL-P) levels correlate strongly with CHD [17] with HDL-P serving as an essential marker of residual risk in both HFrEF and HFpEF [18]. Furthermore, the average size of HDL-P progressively and significantly increases across the spectrum from individuals without HF to those with HFpEF and HFrEF. Interestingly, HDL-P levels are negatively associated with the time to major adverse cardiac event in patients with HF [19].

## 4. HDL Function and Heart Failure

In terms of the molecular mechanisms underlying the impact of lipids on cardiac function, lipids play a crucial role in the short-term metabolic flexibility of the heart. The presence of lipotoxic compounds may be a key factor linking metabolic stress with persistent myocardial tissue damage. In animal models, plasma lipid distribution reflects altered cardiac lipid metabolism leading to HF [20]. As an important component of lipoproteins, HDL can inhibit autophagy and cardiac hypertrophy induced by mechanical stress. HDL maintains the mitochondrial function of cardiomyocytes, reduces oxidative stress, and promotes the uptake of glucose by cardiomyocytes, thus improving the survival rate of cardiomyocytes. HDL induces the production of nitric oxide (NO) by coronary endothelial cells, promotes coronary artery relaxation, and thus increases blood flow perfusion in the failed heart [21]. One study has found that upregulation of HDL inhibits interstitial fibrosis and cardiac remodeling, and prevents HFpEF in mice [22]. In mouse models, overexpressed HDL terminates inflammation and reduces myocardial hypertrophy by eliminating tumor necrosis factor alpha (TNF-α) and interferon gamma (IFN-γ). This results in the improved recovery of left ventricular function after ischemia and reversing pathological remodeling and HFpEF in mice, demonstrating that HDL improves heart failure by removing serum cytokines that may impair heart function [22]. HDL function can be shown by lipid transfer rate, cholesteryl ester transfer protein (CETP), and lecithin cholesterol acyltransferase (LCAT) concentrations. Martinelli *et al*. [23] found a negative correlation between the severity of HF in patients and CETP concentrations. The negative correlation between patients’ CETP concentrations and brain natriuretic peptide (BNP) values further supports the findings of this study by Martinelli and colleagues [23]. Thus, the function of HDL decreases with increasing heart failure disease severity.

## 5. Apolipoproteins of HDL and Heart Failure

HDL is related to many apolipoproteins, including (Apo) A-I and A-II [24]. (Apo) A-I is the main structural component of HDL and plays a critical role as a cofactor of lecithin cholesterol acylase, facilitating the formation of cholesterol esters within HDL particles [25]. Analysis of related studies showed that (Apo) A-I significantly influences HDL’s cardioprotective mechanisms. It has been shown that HDL function is significantly impaired in patients with HF as reflected by HDL inflammatory index (the ability of HDL to inhibit oxidation of oxidized low-density lipoprotein) and paroxysmal oxidase-1 activity. *In vitro*, addition of Apo A-I mimetic peptide 4F partially reversed the HDL inflammatory index [25]. In addition, it has been demonstrated by Mishra M *et al*. [3] that elevated HDL in mice treated with adenovirus-associated serotype 8-human Apo A-I gene during stress overload, resulted in an anti-nutritional effect on the myocardium and an offsetting impact on myocardial pathological remodeling. Together these events led to increased capillary density, and reduced myocardial fibrosis.

Conversely, the impact of (Apo) A-II primarily influences the structural, metabolic, and functional properties of HDL. It enhances the stability of HDL particles, impeding their remodeling, and promoting improved cardioprotective activity. Notably, serum levels of (Apo) A-II serum were found to be negatively associated with 1-year mortality in patients with acute heart failure, suggesting that (Apo) A-II may improve the prognosis of acute heart failure [24].

## 6. PON-1 of HDL and Heart Failure

HDL-associated paraoxonase-1 is an essential mediator of many protective functions of HDL [26]. PON-1 is expressed in a variety of tissues, most commonly in the liver, where it is synthesized and secreted into the blood [27]. Circulating PON-1 is the main detoxification protein, binding to (Apo) A-I in HDL particles. PON-1 inhibits low-density lipoprotein (LDL) oxidation, cholesterol efflux, monocyte binding and transport, plaque inflammation, and destruction of oxidized phospholipids, thereby reducing atherosclerosis and plaque formation. Additionally, by hydrolyzing homocysteine thiolactone, PON-1 may prevent endothelial dysfunction and vascular injury [28]. Notably, a study revealed that heart failure patients with impaired left ventricular systolic function or advanced decompensation exhibited significantly reduced PON-1 activity [15]. This finding underscores the potential relevance of PON-1 in heart failure pathophysiology and suggests its involvement in the impairment of left ventricular function and disease progression.

## 7. Antioxidant Properties of HDL and Heart Failure

Recent studies have found HDL to be an important and significant prognostic factor for the antioxidant defense system in patients with HF [29]. Patients with HF have increased oxidative stress compared to the healthy population. Moreover, disease severity, hospital admissions, and progressive outcomes were more prominent in patients with high oxidative stress [28, 30, 31]. The main reason for oxidative stress is the imbalance between reactive oxygen species (ROS) production and antioxidant defense. Many substances may produce ROS in patients with HF, including mitochondrial electron transport chain (ETC), nicotinamide adenine dinucleotide phosphate (NADPH) oxidase, and other metabolic enzymes. Excessive ROS release can lead to cellular damage through direct effects on proteins and nucleic acids, as well as indirect damage by promoting left ventricular dysfunction through the activation of pro-inflammatory and pro-apoptotic pathways. HDL plays a crucial role in protecting against oxidative stress by removing oxidative factors such as ROS [32]. This antioxidative function of HDL is vital for mitigating the detrimental effects of oxidative stress in HF patients, highlighting its potential therapeutic importance in managing the disease.

## 8. HDL/CRP and Heart Failure

The interplay between inflammation and lipid metabolism has emerged as a prominent area of investigation in cardiovascular diseases. Inflammatory cytokines play a role in modulating the concentration and composition of plasma lipoproteins. In contrast, C-reactive protein (CRP), the most common inflammatory cytokine, and HDL are well-known cardiovascular predictors [33]. Notably, HDL’s anti-inflammatory capacity is independent of many established cardiovascular biomarkers [34]. Recent research has focused on the role of HDL-C as an anti-inflammatory component. However, inflammation has been shown to reduce the concentration and function of HDL-C. In the presence of systemic inflammation, HDL granules contain less cholesterol esters and become larger and richer in free cholesterol, triglycerides, and free fatty acids. As a result, HDL has a lower antioxidant capacity and is less effective at removing cholesterol from cells [33]. CRP and HDL-C play opposing roles in regulating inflammation, thus there may be antagonism between these factors, and their role in influencing future events. In other words, the balance between the pro-inflammatory effects of CRP and the anti-inflammatory properties of HDL-C may influence the course of certain diseases, such as cardiovascular disease, cancer, or all-cause mortality [35]. Inflammation accelerates the process of heart damage, left ventricular dysfunction, and ventricular remodeling, leading to the progression of heart failure. CRP is an important indicator of inflammation, which can predict long-term clinical outcomes and cardiopulmonary status with significant ischemic clinical manifestations in patients with chronic heart failure. Furthermore, CRP is independent of BNP and other indicators [36]. Previous studies have confirmed that systemic inflammation is an important factor leading to acute heart failure. While the factors that trigger this immune activation and systemic inflammatory response are still unclear, the activation of monocytes macrophages and lymphocytes, the renin angiotensin aldosterone system, the sympathetic nervous system, and enhanced intestinal bacterial translocation may be involved in or trigger inflammatory processes [37]. CRP amplifies inflammatory responses through complement activation, which can lead to cardiomyocyte apoptosis and promote ventricular damage or dysfunction. Additionally, CRP directly inhibits the production of NO, which serves as an essential compensatory mechanism for chronic ischemia by promoting angiogenesis [38]. These actions of CRP highlight its involvement in the complex relationship between inflammation and cardiovascular health, and emphasize its potential as a therapeutic target in managing cardiovascular diseases.

Yano *et al*. [39] confirmed that the ratio of HDL to CRP can be used as a marker of inflammatory status in HFpEF patients. In the pathogenesis of HFpEF, systemic microvascular inflammation profoundly impacts myocardial structure and function. HDL-C acts as anti-inflammatory component, while CRP acts as inflammatory component. The HDL/CRP ratio exhibits a clear correlation with disease progression. The high prevalence of comorbidities leads to a systemic inflammatory state, triggering adverse reactions including inflammatory responses to the coronary microvascular endothelium. These pathological changes increase myocardial cell stiffness and interstitial fibrosis, thus accelerating the progression of heart failure [39]. Moreover, the HDL-C/CRP ratio can indicate proper ventricular function. High CRP levels are associated with right ventricular dysfunction. Conversely, in the absence of common factors such as hypertension, diabetes, and cardiovascular disease, a decrease in HDL-C levels can lead to a decline in right ventricular function among patients. Thus, the HDL-C/CRP ratio provides insights into both inflammatory status and ventricular function in HFpEF patients.

## 9. TG/HDL and Heart Failure

Metabolic syndrome, characterized by obesity, hypertriglyceridemia, hypo-HDL cholesterolemia, and hypertension is an important cardiovascular risk factor. One useful marker to identify cardiovascular risk is the triglyceride/HDL-C (TG/HDL-C) ratio. In adults, the TG/HDL-C index has been shown to identify patients with dyslipidemia and insulin resistance [40]. In the phenomenon known as lipotoxicity, there is an excessive accumulation of body lipids that surpasses the storage capacity of adipose tissue, resulting in impaired oxidation of free fatty acids. The impact of increased lipids on the myocardium is profound and multifaceted [41]. In addition, elevated serum triglycerides or lower HDL-C are directly associated with endothelial damage and atherosclerosis. Therefore, it is not difficult to understand that the triglyceride/HDL cholesterol ratio is positively correlated with the severity of heart failure [42]. HF itself may also promote insulin resistance. Neurohumoral activation of HF promotes the metabolism of free fatty acids, which may lead to myocardial and systemic insulin resistance [43]. Earlier studies have shown that patients with HF have a worse prognosis if they have low total cholesterol and triglyceride levels. One explanation may be that low triglyceride and cholesterol levels indicate malnutrition or an advanced stage of HF [7]. Patients with reduced ejection fraction, as well as low baseline totals of cholesterol and triglycerides are at particularly high risk of adverse outcomes when hospitalized for HF [44]. The TG/HDL-C ratio has emerged as a robust predictor of coronary artery disease severity. In a study by Yunke Z *et al*. [45], the association between the TG/HDL-C ratio and in-hospital events of HF was examined. It was observed that patients with higher TG/HDL-C ratios exhibit elevated Gensini scores and more pronounced vascular stenosis. These findings suggest that patients with higher TG/HDL-C ratios are at a heightened risk of developing ventricular remodeling and cardiac dysfunction, emphasizing the clinical significance of this ratio in assessing cardiovascular risk and disease progression.

## 10. Monocyte to HDL Ratio and Heart Failure

A recent study found that patients with chronic heart failure and a monocyte/HDL ratio (MHR) ≥20 had a significant 42% increase in long-term all-cause mortality, underscoring the involvement of inflammation in chronic HF [46]. Zhong JL *et al*. [47] propose that the recruitment and activation monocytes, combined with the infiltration of macrophages are pivotal factors contributing to the development of chronic inflammation, oxidative stress, insulin resistance, and cardiovascular complications in individuals with diabetes. However, HDL-C counteracts the activation of monocytes during inflammatory and oxidative processes, subsequently reducing their activation, adhesion, and inflammatory responses. HDL is considered a protective particle for endothelial cells. HDL stimulates NO release and increases the expression of endothelial NO synthase (eNOS). Furthermore, HDL inhibits the expression of adhesion molecules (e.g., vascular cell adhesion molecules) and suppresses leukocyte adhesion [48]. The HDL-C and (Apo) A-I prevent monocyte activation and monocyte adhesion to the endothelial surface [49]. Recent studies have identified specific mechanisms by which HDL-C molecules affect monocytes by regulating monocyte/macrophage activation, bonding, and migration, thus influencing the proliferation of progenitor cells that can differentiate into monocytes. The anti-inflammatory effects of HDL in macrophages or adipocytes are mainly mediated by cholesterol transport proteins (e.g., adenosine triphosphate (ATP)-binding cassette A-1, ATP-binding cassette G-1, scavenger receptor B-1) and transcriptional regulators. HDL also exhibits antioxidant properties through its association with antioxidant enzymes such as PON1 and platelet- activating factor acetylhydrolase (PAF-AH). HDL inhibits LDL oxidation and intracellular oxidative stress in endothelial cells and scavenges potentially cytotoxic lipids from the circulation. HDL also promotes NO bioavailability in the vasculature by increasing endothelial cell repair and antithrombotic function [50]. These findings underscore the importance of HDL-C as a key player in maintaining cardiovascular homeostasis and its potential as a therapeutic target for managing chronic heart failure and related conditions.

## 11. HDL and the Treatment of Heart Failure

HDL not only influences the development of HF but also holds potential in its treatment. Mishra M *et al*. [3] demonstrated that feeding mice with a diet containing 0.2% cholesterol and 10% coconut oil led to cardiac hypertrophy, impaired cardiac function, and ultimately decreased exercise tolerance. However a two-week treatment intervention with recombinant HDL Milano (MDCO216) resulted in the reversal of cardiac hypertrophy and pathological remodeling, along with the restoration of cardiac function and exercise tolerance. A study by Aboumsallem *et al*. [51] demonstrated that intravenous administration of MDCO-216 improved diastolic function in mice with HF following transection aortic coarctation or sham surgery in 14-week-old mice. Additionally, the treatment reduced interstitial fibrosis and normalized lung weight. These findings suggest that reconstituted HDL may be an effective treatment for HF [51]. A possible mechanism for the electrophysiological role of HDL in HF treatment could be through regulation of cholesterol distribution between raft and non-raft membrane components. Alterations in membrane rafts have the potential to affect the microdomain-specific localization of ion channels, which may drive further alterations in the electrical properties and function of cytomembranes [52]. This has been demonstrated in experiments where the repolarization of cardiomyocytes was shortened in isolated animals with wild-type Apo A-I following administration of recombinant HDL [52]. Additionally, infusion of recombinant HDL shortened the duration of ventricular electrical contractions, as demonstrated by a decreased QT interval corrected for heart rate on the electrocardiogram [52]. In cardiomyocytes isolated *in vitro*, the direct effect of HDL was confirmed by increased phosphorylation of extracellular signal-regulated kinase (ERK) 1/2 to activate transcription factor signal transducer and activator of transcription 3 (STAT3), and by enhanced phosphorylation of protein kinase A (Akt). Finally, the effect of recombinant HDL on cardiac structure further improves cardiac function. Increased capillary density and regression of perivascular fibrosis can enhance cardiac function by improving myocardial microcirculation [52].

## 12. Limitations

With the deepening of HDL research, the understanding of HDL is improving, but many challenging problems remain. Further investigation is needed to understand the mechanism of HDL in the occurrence and development of HF. The role of (Apo) A-I in lipid translocation, and its mechanism of action on the composition, metabolism and function of HDL components remains to be clarified. While the correlation between lipid transfer and cardiovascular disease has been established* in vitro*, there are still gaps our understanding of this important metabolic process, as well as how this knowledge can be translated into clinical practice in HF. Changes in plasma lipoprotein, such as HDL-C, LDL-C, and triglycerides, metabolism during HF have been frequently covered by multiple studies, however the impact of these lipid parameters on disease progression, severity, and prognosis in HF has not been adequately demonstrated. As a treatment method, the pathogenesis and therapeutic effect of recombinant HDL on HF require further experimental research and clinical validation.

## 13. Conclusions

In conclusion, the relationship between HDL and HF is based on its structure and function and is influenced by multiple biomarkers’ interactions. Undoubtedly, it has a protective effect on the heart through antioxidant, anti-inflammatory, anti-apoptotic, and endothelial protective effects. The anti-inflammatory component HDL-C and the inflammatory component CRP constitute the HDL-C/CRP ratio, an indicator of body inflammation status, correlating with ventricular diastolic function in patients with HFpEF. Conversely, an elevated TG/HDL-C ratio can contribute to the development of HF through the effects of high insulin on cardiac function and direct damage to the myocardium from elevated TG. The MHR is a novel biomarker associated with inflammation and oxidative stress, and its association with HF remains intertwined with metabolic syndrome and diabetes mellitus. Its mechanisms still related to the pro-inflammatory and pro-oxidant effects of HDL-C counteracting monocytes. The relationship between high-density lipoprotein and HF and the potential mechanism of this interaction remains to be explored. Nevertheless, the role of HDL as a standard marker of HF is unquestionable.

## Abbreviations

(Apo) A-I, apolipoprotein A-I; Apo M, apolipoprotein M; BNP, brain natriuretic peptide; CHD, coronary heart disease; CRP, C-reactive protein; HDL, high-density lipoprotein; HDL-C, high-density lipoprotein cholesterol; HDL-P, high-density lipoprotein particles; HF, heart failure; HFpEF, heart failure with preserved ejection fraction; HFrEF, heart failure with reduced ejection fraction; LDL, low-density lipoprotein; LDL-C, low-density lipoprotein cholesterol; MDCO216, recombinant HDL Milano; MHR, monocytes/HDL ratio; NO, nitric oxide; PON-1, paraoxonase-1; ROS, reactive oxygen species; S1P, sphingosine-1-phosphate; TG, triglyceride.
